# FDG and Amyloid PET in Cognitively Normal Individuals at Risk for Late-Onset Alzheimer’s Disease

**DOI:** 10.4236/ami.2014.42003

**Published:** 2014-04

**Authors:** John Murray, Wai H. Tsui, Yi Li, Pauline McHugh, Schantel Williams, Megan Cummings, Elizabeth Pirraglia, Lilja Solnes, Ricardo Osorio, Lidia Glodzik, Shankar Vallabhajosula, Alexander Drzezga, Satoshi Minoshima, Mony J. de Leon, Lisa Mosconi

**Affiliations:** 1 New York University School of Medicine, New York, USA; 2 Weill Cornell Medical College, New York, USA; 3 University of Cologne, Cologne, Germany; 4 University of Washington, Seattle, USA

**Keywords:** Alzheimer’s Disease, Early Detection, Positron Emission Tomography, Amyloid Imaging, Glucose Metabolism, Normal Aging

## Abstract

Having a parent affected by late-onset Alzheimer’s disease (AD) is a major risk factor for cognitively normal (NL) individuals. This study explores the potential of PET with ^18^F-FDG and the amyloid- *β* (A*β*) tracer ^11^C-Pittsburgh Compound B (PiB) for detection of individual risk in NL adults with AD-parents.

**Methods:**

FDG− and PiB-PET was performed in 119 young to late-middle aged NL individuals including 80 NL with positive family history of AD (FH+) and 39 NL with negative family history of any dementia (FH−). The FH+ group included 50 subjects with maternal (FHm) and 30 with paternal family history (FHp). Individual FDG and PiB scans were Z scored on a voxel-wise basis relative to modality-specific reference databases using automated procedures and rated as positive or negative (+/−) for AD-typical abnormalities using predefined criteria. To determine the effect of age, the cohort was separated into younger (49 ± 9 y) and older (68 ± 5 y) groups relative to the median age (60 y).

**Results:**

Among individuals of age >60 y, as compared to controls, NL FH+ showed a higher frequency of FDG+ scans vs. FH− (53% vs. 6% p < 0.003), and a trend for PiB+ scans (27% vs. 11%; p = 0.19). This effect was observed for both FHm and FHp groups. Among individuals of age ≤60 y, NL FHm showed a higher frequency of FDG+ scans (29%) compared to FH− (5%, p = 0.04) and a trend compared to FHp (11%) (p = 0.07), while the distribution of PiB+ scans was not different between groups. In both age cohorts, FDG+ scans were more frequent than PiB+ scans among NL FH+, especially FHm (p < 0.03). FDG-PET was a significant predictor of FH+ status. Classification according to PiB status was significantly less successful.

**Conclusions:**

Automated analysis of FDG− and PiB-PET demonstrates higher rates of abnormalities in at-risk FH+ vs FH− subjects, indicating potentially ongoing early AD-pathology in this population. The frequency of metabolic abnormalities was higher than that of A*β* pathology in the younger cohort, suggesting that neuronal dysfunction may precede major aggregated A*β* burden in young NL FH+. Longitudinal follow-up is required to determine if the observed abnormalities predict future AD.

## 1. Introduction

Alzheimer’s disease (AD), the leading cause of dementia in the elderly, is a neurodegenerative disorder with insidious onset and progressive cognitive declines. Many clinical studies indicate that by the time patients come in for diagnosis, too much irreversible brain damage may have already occurred for treatments to be effective. Preventive interventions, once they are developed, ideally would be implemented long before symptoms occur. A major goal in AD research is the detection of biological markers to identify at-risk people at the earliest stages of disease when symptoms are not yet apparent.

PET imaging with 2-[^18^F] fluoro-2-Deoxy-D-glucose (FDG) and amyloid-beta (A*β*) tracers such as ^11^C-Pittsburgh Compound-B (PiB) and other ^18^F-labeled compounds are under investigation as tools to improve the early detection of AD. FDG-PET is routinely used in the early and differential diagnosis of AD and other neurodegenerative disorders, and diagnostic criteria have recently been proposed for amyloid-imaging [1]– [8]. Of relevance to the early detection of AD, characteristic abnormalities of both biomarkers have been observed years prior to clinical decline in asymptomatic, cognitively normal (NL) individuals [9]–[12]. Although A*β* plaques are one of the defining pathological features of AD [13], a large proportion of otherwise healthy, non-demented elderly exhibit substantial A*β* burden [7] [9] [14] [15], making the functional significance of elevated A*β* in this population unclear. FDG-PET abnormalities reflect neuronal dysfunction and correlate well with dementia severity [1] [3] [10], although this biomarker is not as specific to AD. Examination of at-risk individuals represents an ideal way to explore the value of these two imaging modalities in the early detection of AD-typical pathology, prior to cognitive decline.

Apart from age, having a 1^st^ degree family history of AD (FH) is a major risk factor for NL individuals [16] [17]. While the rare early-onset forms of AD have autosomal dominant genetic inheritance, the risk for late-onset AD (LOAD), which comprises over 99% of the AD population after the age of 60, is influenced by several genetic and non-genetic factors. Although LOAD does not show recognizable Mendelian inheritance, risk is to some extent genetically determined, as shown by the familial aggregation of many LOAD cases. Recent biomarker studies showed that NL with LOAD-parents, especially those with an affected mother, manifest an AD-endophenotype characterized by reduced brain metabolism on FDG-PET and increased A*β* deposition on PiB-PET compared to those with negative FH of AD [18]–[21]. Maternal transmission may account for up to 30% of all LOAD cases [22]. These findings suggest that PET may play a role in the early detection of AD in these individuals. However, results were based on group differences and the value of PET to assess AD-like abnormalities on an individual basis in asymptomatic people is unknown. Additionally, there are no published studies that examined individual PET scans in young adults at risk for LOAD. The goal of this study was to examine FDG- and PiB-PET on a subject by subject basis in young to late-middle aged NL individuals with and without a FH of AD.

## 2. Methods

### 2.1. Subjects

This study examined 146 prospectively recruited, clinically and cognitively normal (NL) individuals enrolled in longitudinal PET imaging studies at NYU School of Medicine between 2009 and 2013. These included individuals interested in research participation and risk consultation, self-referred individuals with cognitive complaints, spouses, family members, and caregivers of patients participating in other studies. Subjects received medical, psychiatric, neuropsychological, clinical MRI and PET exams. The study was approved by the NYU IRB. Informed consent was obtained from all subjects. Individuals with medical conditions or history of conditions that may affect brain structure or function, *i.e.* stroke, diabetes, head trauma, any neurodegenerative diseases, depression, hydrocephalus, intracranial mass, and infarcts on MRI, and use of psychoactive medications were excluded. Subjects were 33 – 79 years old, with education ≥12 years, Clinical Deterioration Rating (CDR) = 0, Global Deterioration Scale (GDS) ≤2, Modified Hachinski Ischemia Scale <4 and Mini Mental State Examination (MMSE) ≥26. All subjects had normal cognitive test performance relative to appropriate reference values for age and education. Only individuals who completed both FDG− and PiB-PET procedures and had detailed family history information were included. A FH of LOAD that included at least one 1^st^ degree relative whose AD onset was after age 60 was elicited by using standardized FH questionnaires [19] [21]. All participants were asked to fill in names, dates of birth, age at death, cause of death, and clinical information of all affected family members. The information was confirmed with other family members by interview with the examining neurologist, discussing the parents’ symptomatology and progression of disease. Only individuals whose parents had lived to age ≥65 were included. For those with a FH, the parents’ diagnosis of LOAD was reportedly clinician certified. Subjects were divided into FH positive and negative groups (FH+ vs FH−). We examined parent gender effects by stratifying FH+ subjects into those with maternal (*i.e.*, FHm) and paternal FH (*i.e.*, FHp).

### 2.2. PET Acquisition

Subjects received two PET scans acquired in 3D-mode on an LS Discovery [G.E. Medical Systems, Milwaukee, WI; 5.4 mm FWHM, 30 cm FOV] or a BioGraph PET/CT scanner [Siemens, Knoxville, TN; 1 mm FWHM, 25 cm FOV] following standardized procedures [18]–[20]. Briefly, before PET imaging, an antecubital venous line was positioned for isotope injection. Subjects rested with eyes open and ears unplugged in the quiet and dimly lit scan room. Subjects were positioned in the scanner using laser light beams for head alignment approximately 60 min after injection of 15 mCi of ^11^C-Pittsburgh Compound B (PiB) and scanned for 30 min [18] [19]. The FDG scan procedure started 30 min after the PiB scan or on a separate day. After an overnight fast, subjects were injected with 5 mCi of 2-[^18^F] fluoro-2-Deoxy-D-glucose (FDG), positioned in the scanner 35 min after injection, and scanned for 20 min. Prior to PET, a CT scan was acquired for attenuation correction. All images were corrected for photon attenuation, scatter, and radioactive decay, and reconstructed into a 512 × 512 matrix. The higher resolution scans were degraded to match the resolution of the LS Discovery scans using uniform resolution smoothing parameters [23].

### 2.3. Image Analysis

**Figure 1:**
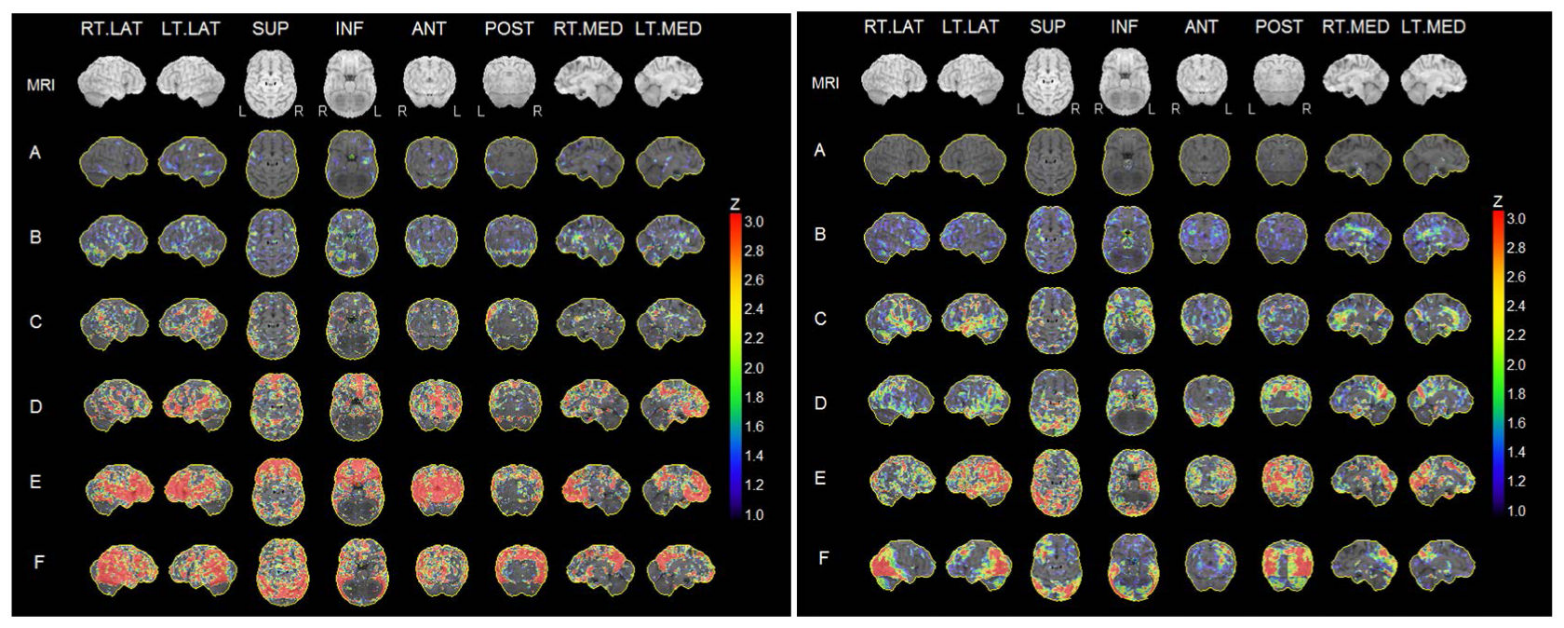
Left panel: Representative FDG-PET patterns in NL individuals: (A, B) FDG−; (C, D) mild hypometabolism of PCC a/o parieto-temporal cortex; (E, F) moderate-to-severe hypmetabolism of PCC a/o parieto- temporal cortex. Right panel: Representative PiB-PET patterns in NL individuals: (A, B) PiB−; (C, D) mild PiB uptakein PCC a/o parieto-temporal cortex; (E, F) moderate-to-severe PiB uptake in PCC a/o parieto- temporal cortex. 3D-SSP maps showing tracer uptake deviations relative to norms are displayed on a color- coded scale and shown on the right and left lateral, superior and inferior, anterior and posterior, right and left medial views of a standardized brain image.

Image analysis was performed blind to clinical data. For each subject, summed PET images corresponding to the 40 – 60 min of FDG data and to the 60 – 90 min of PiB data were generated, and coregistered to their corresponding T1-MRI using a surface-fitting algorithm [24]. Following coregistration, PET scans were processed using the iSSP35-NMP-us standard diagnostic routine of the well-established, rater-independent Neurological Statistical Image Analysis program (NEUROSTAT, University of Washington, Seattle, USA). All scans were realigned to the anterior-posterior commissure line and spatially normalized to the Talairach and Tournoux atlas using an affine transformation with 12 parameters followed by nonlinear warping, yielding a standardized image set with 2.25 mm voxels [3] [25] [26]. The spatially normalized FDG and PiB PET scans of an additional twelve NL individuals (age 42 – 80 yrs, 50% female, education >12 yrs, MMSE ≥ 28, all FH−) with FDG− and PiB− scans were used to generate an FDG and a PiB normative database [25] [26]. PET scans of each subject under study were compared with the corresponding reference database while controlling for pons activity for FDG [27] and for cerebellar uptake for PiB [28] using NEUROSTAT scaling procedures. *Z* scores [Z = (voxel_subject_ − voxel mean_database_)/voxel standard deviation_database_] were calculated on a voxel-basis, and gray matter activities were extracted to predefined surface pixels using a three-dimensional stereotactic surface projection (3D-SSP) technique, which minimizes residual anatomic variances across subjects and partial volume effects, yielding robust voxel- based statistical analysis [3] [25] [26]. 3D-SSP maps allow visualization of deviations in FDG and PiB uptake on an individual basis [3] [10] [11] [25] [26]. Z scores are automatically adjusted by age and gender using scaling procedures [3] [25] [26]. NEUROSTAT generates two Z-score maps for each scan, one depicting negative Z-scores and the other positive Z-scores. Negative Z-score maps were inspected for FDG, and positive Z-score maps for PiB. All 3D-SSP maps were independently inspected by two raters and classified as positive (FDG+, PiB+) or negative (FDG−, PiB−) for presence of a neurodegenerative disease consistent with AD using published protocols with known intra- and inter-rater reliabilities and an absolute Z score threshold of >1.5 SD [3] [4] [10] [29]. The final diagnosis was made by joint agreement. Classification was facilitated by detection of AD-patterns exceeding the predefined Z score threshold within AD-specific regions of interest (ROI) superimposed onto the 3D-SSP maps [3] [10] [30]. ROIs included parietal, temporal, medial and pre-frontal cortex, posterior cingulate cortex (PCC), precuneus, and angular gyrus [1]–[7] [11] [27] [30]. FDG+ scans had focal cortical hypometabolism in PCC, precuneus, parietal, temporal and/or prefrontal cortex of at least one hemisphere, with sparing of sensorimotor, visual cortex basal ganglia and cerebellum [3] [4] [10] [26] [29]. As reduced FDG uptake may occur in frontal cortex in AD, this region was also inspected although frontal hypometabolism alone was not regarded as indicative of AD. FDG− scans had no abnormal findings or had abnormal findings reported other than those meeting the definition of FDG+ (e.g., global decrease in metabolic levels without sparing of sensorimotor and visual cortex and cerebellum; hypometabolism restricted to brain regions not specific to AD) [3] [4] [10] [26] [29]. PiB+ scans had focal cortical PiB uptake in PCC/precuneus, parietal, temporal and/or medial and prefrontal lobes of at least one hemisphere, with sparing of sensorimotor cortex, basal ganglia and cerebellum [1]–[7] [11] [27]. As amyloid deposition may occur in occipital cortex and striatum in AD, these regions were also inspected although PiB uptake in occipital cortex and striatum alone was not regarded as indicative of AD. PiB− scans had no abnormal findings or had abnormal findings reported other than those meeting the definition of PiB+ (e.g., PiB retention restricted to brain regions not specific to AD). The method was further validated vs. visual inspection of raw scans as well as vs. quantitative assessment. As different levels of abnormalities were observed for both tracers, FDG+ and PiB+ scans were further divided into patterns with mild vs moderate-to-severe deficits based on Z scores within diagnostic regions. For both tracers, mild abnormalities were defined by Z ≤ 2.5 and cluster extent >50 voxels, and moderate-to-severe abnormalities by Z > 2.5 and cluster extent >200 voxels (Figure 1).

### 2.4. Statistical Analysis

Statistical analyses were done with SPSS 16.0 (SPSS inc., Chicago, IL). Differences in clinical and demographical measures between groups were examined with *χ*^2^ tests and the general linear model (GLM). *χ*^2^ tests were used to compare the distribution of FDG+ vs FDG−, and PiB+ vs PiB− scans, as well as the degree of biomarkers abnormalities (moderate-to-severe vs mild) between family history groups (FH+ vs FH−), and parent-gender groups (FHm vs FHp). Non-parametric McNemar tests for related samples were used to compare the frequency of FDG+ and PiB+ ratings within groups. To assess the effect of age on diagnostic accuracy, the cohort was separated according to its median age (60 y) into two groups, younger (49 ± 9 y) and older (68 ± 5 y), which were examined in interaction with FH status. Logistic regressions and ROC curves were used to estimate accuracy, sensitivity, specificity, and relative risk (95% confidence interval, C.I.) of individual FDG and PiB scans, and their combination, as risk classifiers. Results were considered significant at p < 0.05.

## 3. Results

### 3.1. Subjects

Of the 146 NL individuals enrolled, 24 were excluded, including 9 subjects who did not complete the FDG or PiB procedure, 4 subjects of age >80 yrs, 4 subjects who were excluded because of comorbidities (*i.e.*, severe depression or MRI abnormalities), and 10 subjects with incomplete family history. The remaining 119 NL individuals were examined in this study (Table 1). Of these, 80 (67%) had a positive family history of AD (FH+), including 50 FHm and 30 FHp. The remaining 39 subjects were FH−. Family history groups were comparable for clinical and demographical measures (Table 1).

### 3.2. PET Findings: Age

**Figure 2:**
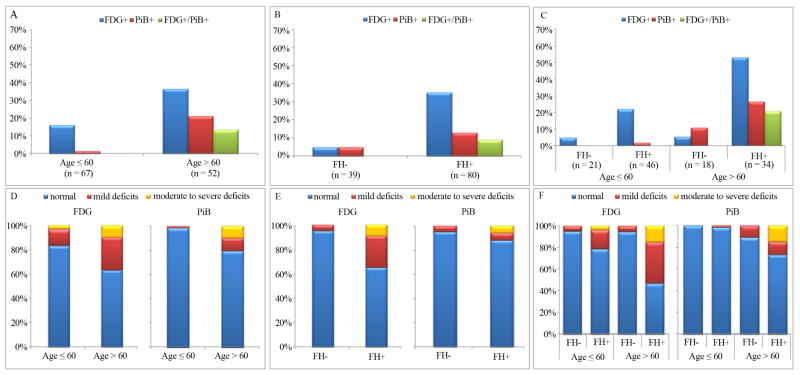
PET abnormalities in NL FH+ vs. FH− individuals. *Top panel:* Percentage of FDG+, PiB+ and FDG+/PiB+ scans by age (A); family history status (B); and age by family history status (C); *Bottom panel:* Percentage of FDG and PiB scans showing absent, mild, or moderate-to-severe abnormalities by age (D); family history status (E), and age by family history status (F).

A significant effect of age was observed for both FDG and PiB-PET. NL of age >60 y showed a higher frequency of FDG+ (37%) compared to those of age ≤60 y (16%; p = 0.01), as well as of PiB+ scans (21% vs 2%; p < 0.001) (Figure 2). Additionally, older individuals showed a higher frequency of FDG+ and PiB+ scans with moderate-to-severe abnormalities compared to younger individuals (p < 0.04; Figure 2).

### 3.3. PET Findings: Family History

**Figure 3:**
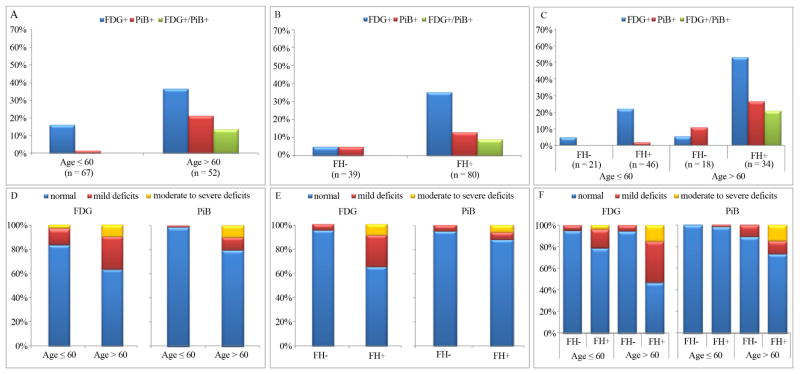
PET abnormalities in NL FHm vs. FHp vs. FH−. *Top panel*: Percentage of FDG+, PiB+ and FDG+/PiB+ scans by parent-gender (A); age by parent-gender status (B); *Bottom panel*: Percentage of FDG and PiB scans showing absent, mild, or moderate-to-severe abnormalities by parent-gender (C); and age by parent-gender status (D).

Across all subjects, FH+ individuals showed a higher frequency of FDG+ scans (28/80, 35%) as compared to FH− (2/39, 5%; p < 0.001), as well as a higher frequency of individuals with moderate-to-severe metabolic deficits (9% vs 0%, respectively, p = 0.002) (Figure 2). There was a non-significant trend towards a higher frequency of PiB+ scans in FH+ vs. FH− (13% vs 5%, p = 0.21, n.s.), and all PiB+ scans with moderate-to-severe abnormalities belonged to the FH+ group (Figure 2). A significant interaction between FH and age was observed for both FDG− and PiB-PET, with FH+ individuals of age >60 y showing the highest frequency of FDG+ and PiB+ scans among all groups (p ≤ 0.001). Among NL of age >60 y, FH+ subjects showed a higher frequency of FDG+ scans (53%), as well as a higher frequency of moderate-to-severe metabolic deficits (15%) compared to FH− (6% and 0%, respectively; p *≤* 0.003). There was a trend towards a higher frequency of PiB+ scans in FH+ vs FH− (27% vs. 11%; p = 0.19). Among NL of age ≤60 y, FH+ subjects showed a trend towards a higher frequency of FDG+ scans vs FH− (22% vs 5%, p = 0.08). There were no group differences for PiB-PET, as only 1 NL subject out of 67 was PiB+. Among FH+ individuals, the frequency of FDG+ scans was higher than that of PiB+ scans in both age cohorts (p ≤ 0.02; Figure 3). No differences between biomarkers were found for the FH− group, at any age.

### 3.4. PET Findings: Parent-Gender Effects

Across all subjects, significant parent-gender effects were observed on FDG–PET. This effect was driven by FHm individuals who showed a higher frequency of FDG+ scans (40%) compared to FH− (5%) and to FHp subjects (27%) (p < 0.001; Figure 3). The FHm group included slightly more subjects with moderate-to-severe metabolic deficits than the other groups (Figure 3). Neither the frequency of PiB+ scans or of moderate-to-severe PiB abnormalities differed between groups (p < 0.35, n.s.), although none of the FH− subjects showed moderate-to-severe PiB abnormalities (Figure 3). A significant interaction between parent-gender FH status and age was observed on both FDG− and PiB-PET (p < 0.005). Among individuals of age >60 y, NL FHm and FHp showed a higher frequency of FDG+ scans compared to FH− (55% and 50% vs 6%, p = 0.003), as well as a higher frequency of moderate to severe deficits (p < 0.02). NL FHm and FHp showed more PiB+ scans than FH− (23% FHm, 33% FHp vs 11% FH−), which did not reach significance (p = 0.33). Among individuals of age ≤60 y, NL FHm showed a higher frequency of FDG+ scans (29%) compared to FH− (5%, p = 0.04) and a trend compared to FHp (11%) (p = 0.07), while the distribution of PiB+ scans was not different between groups (Figure 3). Overall, among FHm individuals, the frequency of FDG+ scans was higher than that of PiB+ scans in both age cohorts (p < 0.03). No differences between biomarkers were found within the FHp group, at any age.

### 3.5. Abnormalities of Both Biomarkers

**Figure 4:**
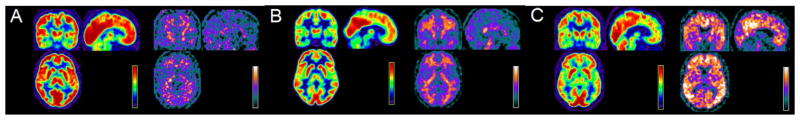
Three representative cases: (A) 50 y/o FH− with FDG−/PiB− scans; (B) 52 y/o FHm with FDG+/PiB− scans; (C) 65 y/o FHm with FDG+/PiB+ scans.

A total of 7 subjects had both FDG+ and PiB+ scans (FDG+/PiB+). All these individuals had age >60 yrs and were FH+, including 4/50 (8%) FHm and 3/30 (10%) FHp (Figure 2 and Figure 3). Three representative cases of different FDG and PiB patterns are shown in Figure 4.

### 3.6. Discrimination Accuracy

#### Family history

Across all subjects, FDG-PET discriminated FH+ vs FH− status with 56% accuracy (35% sensitivity, SS, 85% specificity, SP) and relative risk, RR = 1.6, 95% CI = 1.2 – 1.8 (p = 0.001). Within age groups, FDG-PET was a significant predictor for NL of age >60 y, with 67% accuracy, 53% SS, 94% SP and RR = 2.0, 95% CI = 1.3 – 2.2 (p = 0.003) and showed borderline value for NL of age ≤60 y (45% accuracy, RR = 1.4, 95% CI = 0.9 – 1.6, p = 0.17). PiB–PET did not predict FH status at any age. Adding PiB to FDG in the prediction model did not increase the discrimination accuracy over FDG for any comparisons.

#### Family history parent-gender

Across all subjects, FDG–PET discriminated FHm vs FH− with 64% accuracy (40% SS, 95% SP) and RR = 2.0, 95% CI = 1.4 – 2.3 (p = 0.001). This effect was observed for the older (73% accuracy, RR = 2.5, 95% CI = 1.4 – 3.0, p = 0.003) and younger cohorts (57% accuracy, RR = 1.8, 95% CI = 1.0 – 2.1, p = 0.07). FDG-PET discriminated FHp vs FH− only for NL of age >60 y, yielding 77% accuracy and RR = 3.3, 95% CI = 1.2 – 4.5 (p = 0.02). PiB–PET did not discriminated FHm and FHp groups from controls or from each other and did not add to the prediction accuracy of FDG for any comparisons (p ≥ 0.3).

## 4. Discussion

As several disease-modifying treatments for AD are being evaluated, detection of preclinical brain abnormalities is of great importance to identify individuals at high risk for AD who will most likely benefit from early interventions. By using automated, observer-independent Z scoring software, the present study shows that FDG− and PiB-PET abnormalities are detectable on an individual basis in NL individuals at known increased risk for AD, years prior to possible symptoms onset. NL FH+ showed a higher frequency of metabolic deficits compared to FH−, at any age, whereas increased PiB uptake, reflecting increased fibrillar A*β* deposition, became prominent after age 60 in FH+. The frequency of FDG deficits exceeded that of PiB abnormalities among FH+ individuals, especially those with FHm, of both age cohorts.

Changes in brain histopathology are known to precede the symptoms of AD by many years [31]. According to a popular theoretical model of AD, the “amyloid cascade hypothesis”, A*β* plaques increase during the preclinical phase of AD, causing synapse loss and neuronal death [13]. Other studies have shown that oxidative stress may precede and promote A*β* plaques deposition [32]. While A*β* deposition and metabolic impairments are likely cooccurring phenomena in AD, discrepancies in timing and regional distribution are to be expected, especially in early disease. PiB retention co-localizes with A*β* plaques [5], while FDG uptake reflects local glucose consumption and synaptic functioning, and is therefore influenced by various factors, including reduced synaptic activity [33], neuronal disruption by A*β* oligomers and plaques [13], and disconnection between histopathologically affected regions and functionally associated areas [34] [35]. As such, local A*β* toxicity may not be the only determinant of hypometabolism in early AD.

Fibrillar A*β* deposition was strongly age-related in our data set, as hardly any individuals of age ≤60 y showed significant PiB uptake, whereas 21% of individuals over age 60 had PiB+ scans. These estimates are consistent with other reports showing increased PiB uptake in AD-vulnerable regions of 20% – 50% NL elderly [9] [14] [15] and with post-mortem reports showing that A*β* deposition develops mostly after age 60 [31] [36]. Amyloid deposition was significantly associated with FH status in older individuals, indicating that aging FH+ people are more susceptible to develop brain A*β* compared to FH−. Conversely, hypometabolism on FDG-PET strongly segregated with FH status, especially FHm, irrespective of age. An FDG pattern suggestive of AD was observed in 16% NL of age ≤60 y and 37% NL of age >60 y, the majority of whom were FH+. While there are no prior reports on the prevalence of FDG+ scans in NL individuals, current estimates are quite comparable to those of PiB+ scans in elderly populations [9] [14] [15]. To our knowledge, this is the first study to examine individual PiB or FDG-PET in young adults. The higher prevalence of FDG+ vs. PiB+ scans in our younger FH+ cohort suggests that either metabolic deficits promote, and possibly precede A*β* dysmetabolism in this subset of at-risk individuals, or that FDG reductions are a consequence of A*β* oligomers which are not detectable using PiB.

While we cannot statistically define a temporal or causal relationship between biomarkers due to the cros-ssectional nature of our study and differences in the methods’ sensitivity, biomarkers could be staged as having early or later value for detection of AD risk. For instance, if hypometabolism happens at a higher frequency than A*β* deposition in younger FH+ vs FH− individuals, and yet the two abnormalities occur at the same frequency in older subjects, it may be hypothesized that hypometabolism occurs prior to A*β* deposition. Logistic regressions showed that FDG deficits distinguished FH+ from FH− among older and younger individuals, while PiB failed to do so, especially in the younger cohort. Fibrillar A*β* deposition on PiB-PET may thus be regarded as a “late emerging” biomarker in NL FH+, which is more likely to have changed after “early emerging” hypometabolism on FDG-PET. Future studies are needed to clarify whether metabolic abnormalities in these at-risk individuals are an upstream event to A*β* deposition, or rather reflect disruption of synaptic plasticity by A*β*, in oligomeric or aggregated forms [13] [22]. For practical purposes, present results indicate that FDG-PET may be more informative than PiB-PET for early detection of AD-like changes in NL FH+.

Our findings of hypometabolism in absence of substantial A*β* pathology in young adults FH+, especially those with FHm, are in agreement with reports of metabolic deficits in NL at genetic risk for LOAD [37], and add complexity to current theoretical models of AD progression [9]. These observations are consistent with epidemiological studies showing a main role for maternal transmission in LOAD. Maternal transmission is more frequent than paternal transmission and is associated with a more predictable age of onset and lower performance on cognitive testing in the offspring [22]. Additionally, maternally-inherited LOAD biological endophenotypes are increasingly recognized [18]–[21]. Metabolic changes may be, to some extent, developmental in FHm individuals [22]. It remains to be established whether these changes are due to early, ongoing AD pathology or rather reflect an inborn precondition for later development of disease. Maternal inheritance of oxidative dysmetabolism and other AD-related changes suggests genetic transmission that may be mediated by mitochondrial DNA, which is maternally inherited in humans [22].

Longitudinal follow-ups of our subjects are warranted to determine the predictive value of the observed PET abnormalities. To our knowledge there are no studies that examined PET in the prediction of individual clinical outcome in NL subjects. Therefore, any clinical value at this time is unclear. A few studies of Mild Cognitive Impairment (MCI), a clinical condition at high risk for LOAD, showed high prognostic accuracy for conversion to AD using both FDG− and PiB-PET [4] [10] [29] [38]. About 80% – 90% of MCI with baseline FDG+ or PiB+ scans declined to AD within 1 – 2 years, while the majority of MCI with negative PET scans remained stable over time. By applying similar PET rating criteria as in previous studies, we observed that NL FH+ of age >60 y had 2-fold higher risk of metabolic deficits than controls, which yielded 67% accuracy to discriminate high vs low risk groups. These estimates are necessarily less impressive than in MCI studies, as the conversion rate from normal cognition to AD is substantially lower than for MCI to AD (1% – 2% vs 10% – 30% per year) [39]. Additionally, although FDG-PET abnormalities predict decline from normal cognition to dementia on a group basis (23 – 25), these measures are surrogate markers of AD and doubt remains as to whether the observed hypometabolism is due to AD pathology or other causes.

3D-SSP mapping was developed and extensively validated for FDG-PET [11] [26], and was only recently applied to PiB imaging [40]. 3D-SSP output maps are derived from surface projections. It is possible that, as non-specific PiB uptake is quite elevated in white matter, the program may accidentally project white matter voxels on the surface, increasing the surface area showing abnormalities. This would however result in an increased number of false PiB positives. On the other hand, in severely atrophic brains, the method may underestimate the small amyloid-positive cortical rim surrounding white matter. Partial volume correction (PVC) was not performed in this study because of two considerations. First, our subjects were clinically NL and the oldest was 79, with a median age of 60 y. Atrophic changes severe enough to result in critical underestimation of amyloid burden are more likely in clinical AD patients. Second, it would not be feasible to apply mathematically complex, MRI-based PVC for routine clinical studies. Third, we validated the method against visual reads of all scans and vs. quantitative Z score assessment, showing 100% agreement between 3D-SSP maps and visual inspection of PiB scans. Nonetheless, it remains to be determined whether MRI-based white matter masking would improve the technique’s accuracy, and whether the method may have underestimated detection of very mild, “emerging” PiB abnormalities. Our reference database included carefully selected NL FH− individuals, whose scans were rated as negative for presence of hypometabolism or amyloid pathology, using the same criteria and procedures as with the study cohort. As we further refine the method, larger normative databases may improve detection of subtle abnormalities.

We caution that the NL population selected in our study represents a group with a high a priori risk of preclinical AD-changes, results were made with small numbers of subjects under controlled clinical conditions, and our observations are restricted to NL FH+. Replication of these preliminary research findings in community-based populations is warranted and clinical application is not justified. Nevertheless, we believe that present results are plausible and promising, and set the stage for further studies of asymptomatic individuals at risk for LOAD with longitudinal follow-ups and larger samples. In conclusion, FDG and PiB-PET abnormalities were detectable on an individual basis in asymptomatic people by means of standardized, automated PET analysis procedures, and segregated with FH+ status. This supports the notion that having a 1^st^ degree family history is a major risk factor for LOAD.

## Figures and Tables

**Table 1:** Demographic characteristics by family history group.

Group		Age (y)	Gender (M/F)	Education (y)	MMSE
FH−	Total n = 39	57 (14)	15/24	16 (3)	29 (1)
	Age <60 y, n = 21	47 (12)	7/14	16 (2)	29 (1)
	Age >60 y, n = 18	68 (5)	8/10	16 (4)	29 (2)
FH+	Total n = 80	58 (11)	23/57	17 (2)	29 (2)
	FHp (n = 30)	59 (10)	11/19	17 (2)	29 (1)
	FHm (n = 50)	57 (11)	12/38	17 (2)	29 (2)
	Age <60 y, n = 46	51 (8)	14/32	17 (2)	29 (1)
	FHp (n = 18)	52 (7)	7/11	17 (2)	29 (1)
	FHm (n = 28)	49 (9)	7/21	17 (2)	29 (1)
	Age >60 y, n = 34	68 (4)	9/25	17 (2)	29 (2)
	FHp (n = 12)	69 (5)	4/8	17 (3)	29 (2)
	FHm (n = 22)	68 (4)	5/17	18 (2)	29 (2)

Values are mean (standard deviation).
